# The Impact of Fermented Milk Products Containing *Bifidobacterium longum* BB536 on the Gut Environment: A Randomized Double-Blind Placebo-Controlled Trial

**DOI:** 10.3390/nu16213580

**Published:** 2024-10-22

**Authors:** Ryuta Ejima, Riko Mishima, Akira Sen, Kana Yamaguchi, Eri Mitsuyama, Hiroki Kaneko, Madoka Kimura, Satoshi Arai, Natsumi Muto, Akari Hiraku, Kumiko Kato, Yasuyuki Kuwano, Hiroshi Maruyama, Masahiko Nakamura, Noriyuki Iwabuchi, Manabu Nakano, Toshitaka Odamaki, Miyuki Tanaka

**Affiliations:** 1 Innovative Research Institute, Morinaga Milk Industry Co., Ltd., 1-83, 5-Chome, Higashihara, Zama 252-8583, Kanagawa, Japan; 2 Food Research & Development Institute, Morinaga Milk Industry Co., Ltd., 1-83, 5-Chome, Higashihara, Zama 252-8583, Kanagawa, Japan; 3 Matsumoto City Hospital, 4417-180 Hata, Matsumoto 390-1401, Nagano, Japan

**Keywords:** *Bifidobacterium longum* BB536, probiotics, gut environment, *Faecalibacterium*, tryptophan, tryptophan metabolites

## Abstract

Probiotics, particularly those native to the gut microbiota, have a profound influence on the gut environment. In this study, we conducted a randomized placebo-controlled, double-blind, parallel-group comparison trial to investigate the effects of *Bifidobacterium longum* BB536 (*B. longum* BB536) on the fecal microbiota and metabolite compositions in healthy individuals. We compared the effects of fermented milk produced solely with *Streptococcus thermophiles* and *Lactobacillus bulgaricus* (placebo group) and fermented milk supplemented with *B. longum* BB536 (BY group). Our findings revealed a significantly greater relative abundance of *Faecalibacterium* in the BY group than in the placebo group by the 3rd day, a trend that persisted until the end of the trial on the 17th day. Additionally, the BY group presented significantly increased concentrations of tryptophan (Trp), Indole-3-lactic acid, and Indole-3-aldehyde on the 17th day. A significant positive correlation was observed between the relative abundance of *Faecalibacterium* and the number of viable *B. longum* BB536 bacteria in the feces. The concentrations of Trp and Indole-3-acetic acid were also significantly correlated with the number of viable *B. longum* BB536 bacteria in the feces. Our results suggest that *B. longum* BB536 intake can modulate the gut microbiota and metabolite profiles, which are general indicators for monitoring the gut environment, potentially conferring health benefits to the host.

## 1. Introduction

The human gut is a complex ecosystem that is home to trillions of microorganisms known as the gut microbiota [1]. This ecosystem is crucial for maintaining host health and is intricately involved in various physiological functions, such as intestinal barrier maintenance [2], metabolism [3], immunity [4], and signal transmission in the nervous system [5]. Disruptions in this ecosystem could increase the risk of various diseases, highlighting the importance of maintaining a healthy gut environment [6].

Probiotics, living microorganisms that confer health benefits to the host when consumed in adequate amounts [7], can influence the immune system and intestinal epithelial cells directly and indirectly through the modulation of the gut environment. One of the common sources of these beneficial probiotics is fermented milk, which also possesses beneficial properties such as antioxidant, anti-hypertensive, and anti-allergic effects. These benefits may be attributed to the presence of *Streptococcus thermophiles* and *Lactobacillus bulgaricus*, which are Gram-positive facultative anaerobic and are classified as animal-derived lactic acid bacteria. However, considering the indirect effect by the microbes, species native to the gut microbiota may have a more profound impact on the gut environment due to their enhanced interaction with other gut bacteria. From this perspective, the influence may fundamentally differ between fermented milk containing only species not originally inhabiting the human gut, such as *S. thermophiles* and *L. bulgaricus*, and that which includes species such as *Bifidobacterium*, a part of the gut microbiota.

*Bifidobacterium longum* BB536 (*B. longum* BB536) is a human residential *Bifidobacterium* (HRB) strain that has been safely utilized worldwide for over 50 years. *B. longum* BB536 has been reported to confer various health benefits, including the regulation of the gut environment [8]. Previous studies have reported that the intake of products containing *B. longum* BB536 can regulate the gut environment and bowel movements [9], potentially contribute to immune system regulation [10,11], and reduce visceral and neutral fat levels when it is coingested with *Bifidobacterium breve* MCC1274 [12]. Notably, *B. longum* BB536 is associated with the production of tryptophan metabolites [13], which play an important role in host health. Tryptophan and its metabolite products not only satisfy the nutritional requirements of the host and microorganisms [14] but also contribute to the suppression of intestinal inflammation as ligands for various receptors [15,16,17]. These findings suggest that *B. longum* BB536 could play a pivotal role in maintaining the gut environment through the production of these metabolites. However, few clinical trials have comprehensively reported the effects of consuming fermented milk containing BB536 on both the gut microbiota and these metabolites.

In this study, we conducted a randomized placebo-controlled, double-blind, parallel-group comparison trial (RCT) in healthy individuals, in which traditional fermented milk produced only with *S. thermophiles* and *L. bulgaricus* (placebo group) and fermented milk to which *B. longum* BB536 was added (BY group) were used as test foods. We previously reported the number of viable *B. longum* BB536 bacteria in the feces using this experimental protocol [18]. In the present study, we evaluated the differences in the fecal microbiota and metabolites, which are general indicators for monitoring the environment of the large intestine [19,20], between the BY group and the placebo group in the same experiment, considering short-term (3rd day of intake) and medium-term (17th day of intake) effects. The objective of this study was to elucidate the mechanism by which *B. longum* BB536 confers various beneficial effects to the host.

## 2. Materials and Methods

### 2.1. Clinical Trial Design

This study was a double-blind, randomized, placebo-controlled trial conducted at the Matsumoto Health Laboratory, Japan. The trial aimed to investigate the effect of *B. longum* BB536 on the intestinal environment of healthy adults. The primary outcome of this study was the improvement of the intestinal environment. Changes in gut metabolite abundance and gut microbiota composition served as the outcome indicators. Healthy adults aged 18–64 years were enrolled, with exclusion criteria as previously described [18]. Briefly, participants were randomly assigned to consume either commercial fermented milk containing 20 million CFU/g *B. longum* BB536 or an identical placebo daily for a 17-day intervention period following a 1-week preobservation period. All dietary intake during the intervention was recorded with the food-recording app Calomeal^®^ (Life Log Technology, Inc., Tokyo, Japan). Compliance was monitored by checking the remaining quantity of fermented milk and app entries. The analyses of the per-protocol set that were performed in this study were the same as those of the participants described in a previous study [18], and the per-protocol set was composed of 17 participants in the placebo group and 17 participants in the BY group. Fecal samples were collected on specific days using Raku-Ryu cups, transferred to a specific collection tube, enclosed in an AneroPouch Kenki (Mitsubishi Gas Chemical Co., Tokyo, Japan), and transported to the laboratory within 24 h. This trial was registered at the University Hospital Medical Research Network under UMIN000052110.

### 2.2. Bacterial Strains and Culture Conditions

The *B. longum* BB536 frozen culture was obtained from Morinaga Milk Industry (Tokyo, Japan), and *F. prausnitzii* JCM 39207 was purchased from the Japan Collection of Microorganisms (Tsukuba, Japan). All the strains used in this study were precultured in YCFA medium [21] under anaerobic conditions at 37 °C for 24 h. To evaluate the effect of *B. longum* BB536 on *F. prausnitzii* JCM 39207, the strains (1 × 10^6^ CFU/mL for both) were cultured under anaerobic conditions at 37 °C for 24 h in Gifu anaerobic medium broth (Shimadzu Diagnostics Co., Ltd., Kyoto, Japan).

### 2.3. 16S rRNA Gene Analysis

Bacterial DNA was extracted from the sample and amplified as previously described [21]. The V3-V4 region of the bacterial 16S rRNA gene was paired-end sequenced using the Illumina NextSeq 1000 platform with a NextSeq 1000/2000 P1 reagent kit (600 cycles) (Illumina, Inc., San Diego, CA, USA). The sequences were analyzed via QIIME2 (version 2022.8) [22]. The demultiplexed reads were processed via the following steps: filtering, denoising, merging, chimera removal, and generating the amplicon sequence variants (ASVs) via DADA2 [23]. The ASVs were taxonomically assigned on the basis of the Greengeens2 database (version 2022.10).

### 2.4. Faecalibacterium-Specific Quantitative PCR Analysis

Quantitative PCR analysis was performed by using a CFX96 Thermocycler (Bio-Rad Laboratories, Hercules, CA, USA) with a real-time PCR detection kit (Clostridium cluster iv: RI-0001) (TechnoSuruga Laboratory Co., Ltd., Nagasaki, Japan) and TB Green Premix Ex Taq II (TaKaRa Bio Inc., Shiga, Japan) according to the manufacturer’s instructions.

### 2.5. Metabolomic Analysis of Fecal Metabolites

#### 2.5.1. Chemicals and Reagents

All chemicals used for target analysis, including internal standards, were purchased from Tokyo Chemical Industry Co., Ltd. (Tokyo, Japan), Sigma-Aldrich (St. Louis, MO, USA), and FUJIFILM Wako Pure Chemical Corporation (Osaka, Japan) (Tables S1 and S2). LCMS-grade water, methanol, and formic acid were purchased from FUJIFILM Wako Pure Chemical Corporation. The mobile phase and pH buffering solution were specifically purchased from SHIMADZU GLC Ltd. (Kyoto, Japan).

#### 2.5.2. Extraction of Metabolites from Fecal Samples

Approximately 100 mg (±5 mg) of each fecal sample was suspended in 1000 μL of IS solution (1 μM MO in 50% water/methanol) or water. These suspensions were homogenized with 0.1 mm zirconia/silica beads by vigorous shaking (25 Hz, 10 min) via a TissueLyser instrument and centrifuged at 20,380× *g* for 5 min. The supernatants were then transferred into NANOSEP 3K OMEGA (Cytiva, Marlborough, MA, USA) or ULTRAFREE MC PLHCC 5K (Human Metabolome Technologies, Inc., Yamagata, Japan) media. The supernatants transferred to the ultrafiltration filters were then centrifuged at 9100× *g* for 3–5 h. The filtered samples were stored at −80 °C until further analysis.

#### 2.5.3. Liquid Chromatography–Tandem Mass Spectrometry (LC-MS/MS) Analysis

LC-MS/MS analysis was performed using a Nexera X2 system (Shimadzu, Kyoto, Japan) equipped with two LC-40D XR pumps, a DGU-405 degasser, a SIL-40C XR autosampler, a CTO-40C column oven, and a CBM-40 control module coupled with an LC-8045 triple quadrupole mass spectrometer (Shimadzu). An XBridge^®^ C8 column (4.6 × 150 mm, 5 μm; Waters Corporation, Milford, MA, USA) was used for the separation of metabolites. The mobile phase was composed of A: 0.1% (*v*/*v*) formic acid in water and B: 0.1% (*v*/*v*) formic acid in methanol. The flow rate, column temperature, and injection volume were set as 0.2 mL/min, 40 °C, and 2 µL, respectively. The gradient program for mobile phase B was as follows: 0 min, 30%; 5 min, 30%; 42 min, 95%; 47 min, 95%; 50.5 min, 30%; and 60 min, 30%. The mass spectrometer was equipped with an electrospray ionization (ESI) source under the following conditions: nebulizing gas flow, 3 L/min; heating gas flow, 10 L/min; interface temperature, 300 °C; desolvation line temperature, 250 °C; heating block temperature, 400 °C; drying gas flow, 10 L/min; and collision-induced dissociation gas pressure, 230 kPa.

#### 2.5.4. HPLC Analysis

The HPLC analysis was performed using a Nexera X2 system (Shimadzu) equipped with two LC-40D pumps, a DGU-405 degasser, a SIL-40C autosampler, a CTO-40C column oven, a CBM-40 control module, and a CDD-10A VP conductivity detector. A tandem ion exclusion column (Shim-pack SCR-102H, 300 mm × 8.0 mm, 7 µm; SHIMADZU GLC Ltd.) was used for the separation of metabolites. The mobile phase and the pH buffering solution were delivered at a flow rate of 0.8 mL/min. The column temperature and injection volume were set as 50 °C and 10 µL, respectively.

### 2.6. Statistical Analysis

Intergroup differences in the fecal microbiota on days 3 and 17 were analyzed with Quade’s nonparametric analysis of covariance (Quade’s nonparametric ANCOVA) after centered log-ratio (CLR) transformation. Similarly, intergroup differences in fecal metabolites on days 3 and 17 were analyzed with Quade’s nonparametric ANCOVA. Other outcomes were compared between groups with Student’s *t* test and the Mann–Whitney U test, as appropriate. Correlation analysis was performed via Spearman’s correlation coefficient. Statistical analyses were performed via IBM SPSS Statistics (ver. 29.0.2.0 (20); Armonk, NY, USA), except Spearman’s correlation analysis, which was conducted via R (ver. 4.3.1). When data were missing, we performed listwise deletion to handle these missing values. A *p* value of <0.05 was considered statistically significant.

### 2.7. Data Availability

The raw sequence data have been deposited in the DNA Data Bank of Japan (DDBJ) Sequence Read Archive (DRR596307-DRR596404) under BioProject no. PRJDB18808. This project includes links and access to fecal sample data (BioSample SAMD00819537-SAMD00819634).

## 3. Results

### 3.1. Differences in Fecal Microbiota Composition Between the BY and Placebo Groups

**Figure 1:**
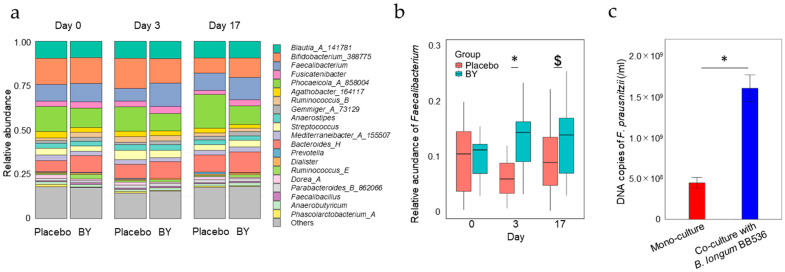
Impact of *B. longum* BB536 bacteria on the fecal microbiota composition: (**a**) stacked bar graph showing the 20 most abundant genera in the fecal microbiota; (**b**) the relative abundance of *Faecalibacterium* in fecal samples; (**c**) DNA copy number of *F. prausnitzii* JCM 39207 when cocultured with *B. longum* BB536 in GAM medium at 37 °C for 24 h. Statistical significance is indicated as follows: $ *p* < 0.1; * *p* < 0.05 with (**b**) Quade’s nonparametric ANCOVA and (**c**) Student’s *t* test.

We evaluated the impact of milk fermented with *B. longum* BB536 (BY) on the fecal microbiota via 16S rRNA gene sequencing analysis of fecal samples collected before intake and on days 3 and 17. The alpha diversity of the fecal microbiota was compared between groups using Chao1, observed features, Pielou’s evenness, and Faith’s phylogenetic diversity metrics as scoring metrics. However, no significant disparities were observed between the placebo and BY groups (Figure S1). We then compared the relative abundance at the genus level. Compared with the placebo group, the BY group presented a significantly greater relative abundance of *Faecalibacterium* on day 3, and this trend was still observed on day 17 (Figure 1a,b). Additionally, significant differences in the abundances *Phascolarctobacterium*_A on days 3 and 17 and *Holdemanella* on day 17 were also observed between the two groups (Tables S3 and S4). Intriguingly, on day 3, the relative abundance of *Faecalibacterium* was positively correlated with the number of viable *B. longum* BB536 bacteria detected in the fecal samples on the same day (Figure S2). To validate the beneficial relationship, *B. longum* BB536 and a representative *Faecalibacterium*, *Faecalibacterium prausnitzii*, were cocultured. The results indicated that compared with monoculture with *F. prausnitzii* alone, coculture with *B. longum* BB536 significantly promoted the growth of *F. prausnitzii* (Figure 1c).

### 3.2. Differences in Fecal Metabolite Abundance Between the BY and Placebo Groups

**Figure 2:**
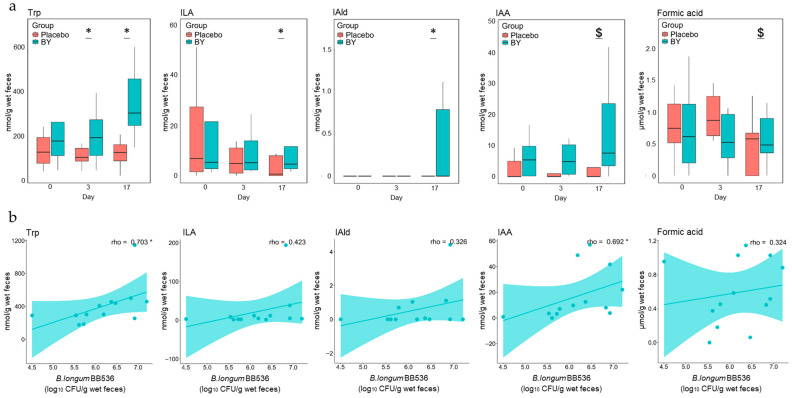
Correlation between fecal metabolite concentrations and viable *B. longum* BB536 counts: (**a**) quantitative measurements of fecal Trp, IAA, ILA, IAld, and formic acid concentrations; (**b**) correlation of the concentrations of the same five metabolites on day 17 and the viable *B. longum* BB536 count. Statistical significance is indicated as follows: $ *p* < 0.1; * *p* < 0.05; with (**a**) Quade’s nonparametric ANCOVA and (**b**) Spearman’s correlation.

**Figure 3:**
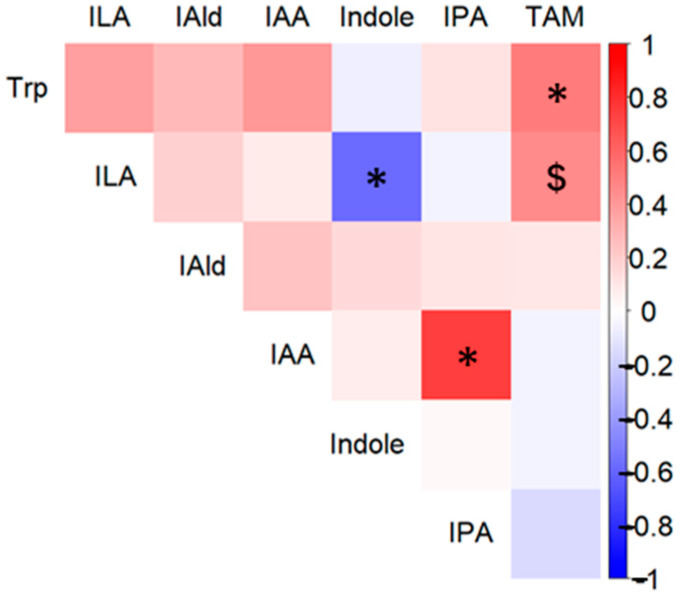
Heatmap of Spearman’s correlations among Trp metabolites on day 17. The map displays pairwise comparisons of Trp, ILA, IAA, IAld, Indole, Indole-3-propionic acid (IPA), and tryptamine (TAM) on day 17. Statistical significance is indicated as follows: $ *p* < 0.1; * *p* < 0.05.

We evaluated the impact of BY intake on the abundance of fecal metabolites, short-chain fatty acids (SCFAs), and tryptophan (Trp) metabolites. Fecal samples were collected before intake and on days 3 and 17 of the experiment and analyzed via HPLC and LC-MS/MS. Sixteen types of metabolites were detected in both the BY and placebo groups, five of which exhibited different concentrations (Table S5). Trp, Indole-3-lactic acid (ILA), and Indole-3-aldehyde (IAld) were present at significantly higher concentrations in the BY group than in the control group on day 17, and Indole-3-acetic acid (IAA) also tended to be present at higher concentrations (Figure 2a). The concentration of formic acid in feces tended to be lower in the BY group than in the placebo group on day 17. Spearman’s correlation analysis was conducted between the five metabolites with significant differences or trends between groups on day 17 and the number of viable *B. longum* BB536 bacteria detected in feces. This analysis revealed that the concentrations of Trp and IAA in feces were significantly positively correlated on day 17 (Figure 2b). Although significant correlations among different Trp metabolites were not detected, the concentration of several metabolites demonstrated a positive correlation with that of Trp (Figure 3). Interestingly, a significant negative correlation was observed between indole and ILA. Conversely, no significant differences were observed in dietary nutrient intake during the trial period between the two groups on the basis of dietary records (Table S6). Furthermore, the levels of most Trp metabolites did not significantly differ between the BY and placebo milk products. However, Trp, which showed the most notable difference, demonstrated a significantly lower concentration in the BY-enriched milk product, a finding that is intriguing given its inverse relationship with fecal Trp concentrations (Figure S3).

### 3.3. Correlations Between the Number of B. longum BB536 Bacteria That Survived Digestion and the Diversity of the Fecal Microbiota

**Figure 4:**
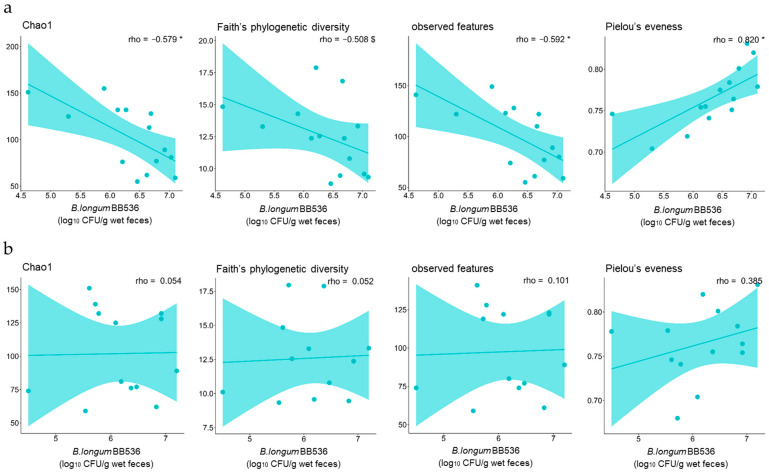
Correlation between alpha diversity of the fecal microbiota and viable *B. longum* BB536 count. Correlation of preintake fecal microbiota alpha diversity with viable *B. longum* BB536 count on (**a**) day 3 and (**b**) day 17. Alpha diversity was scored by Chao1, Faith’s phylogenetic diversity, observed features, and Pielou’s evenness tests. Statistical significance is indicated as follows: $ *p* < 0.1; * *p* < 0.05, with Spearman’s correlation.

Given the significant correlations observed between the abundances of certain gut bacteria or the concentrations of certain metabolites and the number of surviving *B. longum* BB536 bacteria in feces, it is hypothesized that the ability of these bacteria to survive in the gut is crucial for their beneficial effect on the gut environment. Therefore, to elucidate the factors affecting the survival of bacteria in the gut environment, a correlation analysis was conducted between preintake information from the study participants and the number of viable *B. longum* BB536 bacteria detected in feces. The results showed that the number of viable *B. longum* BB536 bacteria detected in feces on day 3 was significantly negatively correlated with Chao1, observed features, and Faith’s phylogenetic diversity and significantly positively correlated with Pielou’s evenness (Figure 4a). Conversely, no significant correlations were detected between the number of viable *B. longum* BB536 detected in feces on day 17 and any of the scores (Figure 4b).

## 4. Discussion

The association between probiotic intake and host health has garnered considerable attention in recent years [24]. Probiotics can influence the host directly by acting on immune cells [25] and modifying the gut microbiota composition [26] and indirectly by regulating metabolite abundance [27]. The strain *B. longum* BB536, used in our study, has been reported to impact the host immune function and gut microbiota composition [28]. However, the specific benefits to the host from fermented milk containing this strain, compared with general fermented milk using only lactic acid bacteria, remain to be fully elucidated. In this study, we conducted an RCT in which study participants consumed fermented milk containing only lactic acid bacteria or fermented milk fortified with *B. longum* BB536 and the effects on both the fecal microbiota and metabolite profiles were assessed. These elements serve as general indicators for monitoring the gut environment. Our findings underscore the impact of consuming BY on the gut environment, as evidenced by significant changes in these indicators.

Compared with the placebo group, the BY group presented an increased relative abundance of *Faecalibacterium* on day 3 (Figure 1a,b), and in vitro assays revealed an increase in *F. prausnitzii* in the presence of *B. longum* BB536 (Figure 1c). As *Faecalibacterium* has been reported to proliferate by utilizing acetic acid [29], it is plausible that the acetic acid produced by *B. longum* BB536 facilitated the growth of *Faecalibacterium*. The butyric acid produced by *Faecalibacterium* not only functions as a vital energy source for colon cancer cells [30] but has also been reported to exhibit anti-inflammatory effects [31], bolster intestinal wall integrity [32], and confer protection against colon cancer [33]. However, no significant difference in the fecal concentration of butyric acid in the feces was detected between the two groups (Table S5). This could be attributed to insufficient diet-derived nutrients for *Faecalibacterium* to produce butyric acid. Additionally, given that blood SCFAs are reportedly more sensitive to probiotic intake than are fecal SCFAs, the amount of SCFAs absorbed by the host may vary, leading to different blood concentrations [34]. The relative abundance of *F. prausnitzii* reportedly decreases in patients with inflammatory bowel disease (IBD) and colon cancer [35], and we postulate that maintaining a high proportion of *Faecalibacterium* itself may play a pivotal role in preserving host health. However, further investigations are warranted to examine the effects of probiotics on the production of SCFAs.

Among the substances produced by gut microbes, Trp metabolites are of paramount importance. The gut microbiota is believed to contribute to host health by converting Trp into various metabolites [36]. These metabolites function as ligands for aryl hydrocarbon receptors (AhRs) and hydroxycarboxylic acid receptor 3 (HCA3), which play crucial roles in maintaining host homeostasis [37]. In this study, we observed that, compared with the placebo group, the BY group presented elevated fecal concentrations of Trp metabolites such as ILA, IAA, and IAld on day 17 (Figure 2a). ILA, produced by *Bifidobacterium*, has been reported to play an important role in maintaining infant health by decreasing Th2, Th17, and IFN-β production, thereby contributing to the suppression of intestinal inflammation [38,39]. The fecal concentration of IAA is known to decrease in patients with IBD and alcoholic hepatitis [40,41]. IAld has been reported to potentially play a role in suppressing *Candida albicans* infection induced by IL-22 [42] and enhancing intestinal barrier function [43].

Notably, the Trp concentration was significantly greater in the BY group than in the placebo group on day 3, and this difference became more pronounced on day 17 (Figure 2a). Trp may be absorbed from the colon via the large neutral amino acid transporter small subunit 2 (LAT2) to meet the nutritional requirements [44] of the host. Trp is reportedly converted into various biological signaling molecules through two major metabolic pathways in the host, namely, the kynurenine pathway and the serotonin pathway [45]. Kynurenine, produced by the kynurenine pathway, has been reported to regulate the activity of immune cells and to exhibit anti-inflammatory effects [46]. Conversely, serotonin and 5-hydroxy tryptophan, which are produced via the serotonin pathway, function as neurotransmitters and play crucial roles in regulating sleep [47]. They have also been associated with mental disorders such as depression and anxiety [48]; therefore, our findings may support a previous study that reported that *B. longum* BB536 may act as a psychobiotic [49].

In this study, we observed that the high concentrations of Trp metabolites may have been produced by gut microbes that naturally inhabit the gut metabolizing the increased Trp from *B. longum* BB536 supplementation. Many previous studies have demonstrated that gut microbes can convert Trp into various Trp metabolites. For example, IAA is reportedly produced by a variety of bacteria, including *Bifidobacterium*, *Bacteroides*, *Clostridium*, *Parabacteroides*, and *Eubacterium*, while IAld is also reported to be produced by *Lactobacillus*, among others [50]. Indeed, the fecal culture results from a study using a human digestive tract model have indicated that Trp supplementation increases the concentration of Trp metabolites [51]. Conversely, the mechanism by which probiotic intake increases the concentration of Trp in feces remains elusive. A previous report demonstrated that the intake of *B. breve* CCFM1025 significantly increased the concentration of Trp in the feces, but the underlying mechanism remains unclear [52]. We hypothesized that the differences in the diet or the consumed test food may have an effect, but no difference was observed in the diet of either group in this study (Table S6). In contrast, Trp was present at significantly lower concentrations in the fermented milk supplemented with *B. longum* BB536 (Figure S3). Previous reports have indicated that gut bacteria maintain a symbiotic relationship in the gut by complementing each other’s nutrient needs [53]. In particular, the results of an in silico analysis revealed that Trp contributes to the diversity and homeostasis of the microbiota as a nutrient for gut bacteria [14]. On the basis of these previous studies, it is plausible that Trp accumulates only under certain conditions where *B. longum* BB536 coexists with other gut bacteria. Nonetheless, these are merely hypotheses, and further research is warranted in the future.

Previous in vitro studies have suggested that HRB converts indole to ILA [13]. However, in this study, no significant difference in indole content was detected between the two groups (Table S5). Nevertheless, the significant negative correlation observed between the concentrations of indole and ILA on day 17 (Figure 3) implies that the conversion from indole to ILA occurred. The absence of a significant difference may be attributed to the large disparity in the concentration of Trp or the presence of different indole producers between the groups.

In this study, a positive correlation was observed between some of the gut bacteria and metabolites that differed in abundance between the two groups and the number of viable *B. longum* BB536 bacteria detected in feces (Figure 2b and Figure S2). Therefore, it is highly plausible that identifying factors that affect the number of live bacteria reaching the gut can enhance the beneficial effects of *B. longum* BB536 intake on the gut environment. We observed that the number of viable *B. longum* BB536 bacteria that reached the gut after 3 days was related to the alpha diversity of the fecal microbiota before intake (Figure 4a). The negative correlation with Chao1, observed features, and Faith’s phylogenetic diversity indicates that greater species diversity decreases the percentage of viable bacteria that reach the gut, whereas a positive correlation with Pielou’s evenness suggests that an even distribution of species increases this percentage. These findings suggest that the greater the diversity of the gut microbiota is, the more challenging it is for newly arrived *B. longum* BB536 to survive in the gut. In fact, similar phenomena have been confirmed in reports on environmental bacteria [54]. Conversely, the number of live *B. longum* BB536 bacteria reaching the gut on day 17 did not have a notable relationship with this diversity (Figure 4b). Therefore, it is predicted that the richness and evenness of species in the gut microbiota are important factors in determining the number of viable *B. longum* BB536 bacteria reaching the gut and that their influence diminishes as the intake period is extended.

This study, while providing valuable insights, has several limitations. First, the trial included only 34 subjects who met the criteria across both groups. To obtain more robust data, expanding the sample size in future clinical trials would be beneficial. Second, the mechanism underlying the observed increase in Trp concentration in the BY group in this study remains unclear. To elucidate this phenomenon, further verification experiments are needed to elucidate the intricate crosstalk between bacteria. By addressing these challenges, we can shed more light on the complex effects of probiotics, including *B. longum* BB536, and contribute to the development of more effective probiotic products.

## 5. Conclusions

In conclusion, our study demonstrated that the intake of milk products containing *B. longum* BB536 bacteria led to an increase in the relative abundance of *Faecalibacterium*, a key indicator of gut health, by the 3rd day of intake. Furthermore, a significant alteration in the concentration of Trp metabolites, which function as signaling molecules and serve as another general indicator for monitoring gut health, was observed in the fecal samples on day 17. These changes, which were more pronounced with a greater number of viable *B. longum* BB536 bacteria present in the fecal samples, suggest an improvement in gut health following BY intake. We posit that these findings lend support to the notion that the regular intake of *B. longum* BB536 bacteria can contribute to overall health by regulating the gut environment.

## Data Availability

The data collected in this study can be found in the published article.

## Supplementary Materials

The following supporting information can be downloaded at: https://www.mdpi.com/article/10.3390/nu16213580/s1, Figure S1: Alpha diversity indices of the fecal microbiota; Figure S2: Correlation of CLR-transformed abundance of *Faecalibacterium* and viable *B. longum* BB536 count on day 3; Figure S3: Concentrations of Trp, IAA, ILA, and IAld in fermented milk. * *p* < 0.05, with Student’s *t* test; Table S1: List of Trp metabolites and internal standards for LC-MS/MS; Table S2: List of SCFAs for HPLC; Table S3: Comparison of the relative abundances of the top 50 bacterial genera on day 3; Table S4: Comparison of the relative abundances of the top 50 bacterial genera on day 17; Table S5: Comparison of the concentrations of metabolites in feces; Table S6: Comparison of estimated total nutrient intake during the trial period.

## References

[1] Daniel N., Lécuyer E., Chassaing B. (2021). Host/microbiota interactions in health and diseases-Time for mucosal microbiology!. Mucosal Immunol..

[2] Di Tommaso N., Gasbarrini A., Ponziani F.R. (2021). Intestinal barrier in human health and disease. Int. J. Environ. Res. Public Health.

[3] Bohan R., Tianyu X., Tiantian Z., Ruonan F., Hongtao H., Qiong W., Chao S. (2019). Gut microbiota: A potential manipulator for host adipose tissue and energy metabolism. J. Nutr. Biochem..

[4] Yoo J.Y., Groer M., Dutra S.V.O., Sarkar A., McSkimming D.I. (2020). Gut microbiota and immune system interactions. Microorganisms.

[5] Margolis K.G., Cryan J.F., Mayer E.A. (2022). The microbiota-gut-brain axis: From motility to mood. Gastroenterology.

[6] Levy M., Kolodziejczyk A.A., Thaiss C.A., Elinav E. (2017). Dysbiosis and the immune system. Nat. Rev. Immunol..

[7] Markowiak P., Śliżewska K. (2017). Effects of probiotics, prebiotics, and synbiotics on human health. Nutrients.

[8] Wong C.B., Odamaki T., Xiao J.-Z. (2020). Insights into the reason of Human-Residential Bifidobacteria (HRB) being the natural inhabitants of the human gut and their potential health-promoting benefits. FEMS Microbiol. Rev..

[9] Nakamura Y., Suzuki S., Murakami S., Nishimoto Y., Higashi K., Watarai N., Umetsu J., Ishii C., Ito Y., Mori Y. (2022). Integrated gut microbiome and metabolome analyses identified fecal biomarkers for bowel movement regulation by *Bifidobacterium longum* BB536 supplementation: A RCT. Comput. Struct. Biotechnol. J..

[10] Odamaki T., Xiao J.-Z., Iwabuchi N., Sakamoto M., Takahashi N., Kondo S., Miyaji K., Iwatsuki K., Togashi H., Enomoto T. (2007). Influence of *Bifidobacterium longum* BB536 intake on faecal microbiota in individuals with Japanese cedar pollinosis during the pollen season. J. Med. Microbiol..

[11] Li Y., Arai S., Kato K., Iwabuchi S., Iwabuchi N., Muto N., Motobayashi H., Ebihara S., Tanaka M., Hashimoto S. (2023). The potential immunomodulatory effect of *Bifidobacterium longum* subsp. longum BB536 on healthy adults through plasmacytoid dendritic cell activation in the peripheral blood. Nutrients.

[12] Sato S., Arai S., Kato K., Yoshida K., Iwabuchi N., Sagami T., Tanaka M. (2024). Effects of *Bifidobacterium longum* BB536 and *Bifidobacterium breve* MCC1274 on body composition in normal and overweight adults in randomized placebo-controlled study. Nutrients.

[13] Yong C.C., Sakurai T., Kaneko H., Horigome A., Mitsuyama E., Nakajima A., Katoh T., Sakanaka M., Abe T., Xiao J.-Z. (2024). Human gut-associated *Bifidobacterium* species salvage exogenous indole, a uremic toxin precursor, to synthesize indole-3-lactic acid via tryptophan. Gut Microbes.

[14] Starke S., Harris D.M.M., Zimmermann J., Schuchardt S., Oumari M., Frank D., Bang C., Rosenstiel P., Schreiber S., Frey N. (2023). Amino acid auxotrophies in human gut bacteria are linked to higher microbiome diversity and long-term stability. ISME J..

[15] Sun M., Ma N., He T., Johnston L.J., Ma X. (2019). Tryptophan (Trp) modulates gut homeostasis via aryl hydrocarbon receptor (AhR). Crit. Rev. Food Sci. Nutr..

[16] Illés P., Krasulová K., Vyhlídalová B., Poulíková K., Marcalíková A., Pečinková P., Sirotová N., Vrzal R., Mani S., Dvořák Z. (2020). Indole microbial intestinal metabolites expand the repertoire of ligands and agonists of the human pregnane X receptor. Toxicol. Lett..

[17] Sakurai T., Horigome A., Odamaki T., Shimizu T., Xiao J.-Z. (2021). Production of hydroxycarboxylic acid receptor 3 (HCA_3_) ligands by *Bifidobacterium*. Microorganisms.

[18] Sen A., Kimura M., Ejima R., Arai S., Mitsuyama E., Kaneko H., Mishima R., Muto N., Hiraku A., Kato K. (2024). Assessing probiotic viability in the gastrointestinal tract in randomized placebo controlled trial: A combined approach of molecular biology and novel cultivation techniques. Benef. Microbes.

[19] Martín R., Rios-Covian D., Huillet E., Auger S., Khazaal S., Bermúdez-Humarán L.G., Sokol H., Chatel J.-M., Langella P. (2023). *Faecalibacterium*: A bacterial genus with promising human health applications. FEMS Microbiol. Rev..

[20] McCann J.R., Rawls J.F. (2023). Essential amino acid metabolites as chemical mediators of host-microbe interaction in the gut. Annu. Rev. Microbiol..

[21] Murakami R., Hashikura N., Yoshida K., Xiao J.-Z., Odamaki T. (2021). Growth-promoting effect of alginate on *Faecalibacterium prausnitzii* through cross-feeding with Bacteroides. Food Res. Int..

[22] Bolyen E., Rideout J.R., Dillon M.R., Bokulich N.A., Abnet C.C., Al-Ghalith G.A., Alexander H., Alm E.J., Arumugam M., Asnicar F. (2019). Reproducible, interactive, scalable and extensible microbiome data science using QIIME 2. Nat. Biotechnol..

[23] Callahan B.J., McMurdie P.J., Rosen M.J., Han A.W., Johnson A.J.A., Holmes S.P. (2016). DADA2: High-resolution sample inference from Illumina amplicon data. Nat. Methods.

[24] Sánchez B., Delgado S., Blanco-Míguez A., Lourenço A., Gueimonde M., Margolles A. (2016). Probiotics, gut microbiota, and their influence on host health and disease. Mol. Nutr. Food Res..

[25] Galdeano C.M., Cazorla S.I., Dumit J.M.L., Vélez E., Perdigón G. (2019). Beneficial effects of probiotic consumption on the immune system. Ann. Nutr. Metab..

[26] Wieërs G., Belkhir L., Enaud R., Leclercq S., de Foy J.-M.P., Dequenne I., de Timary P., Cani P.D. (2020). How probiotics affect the microbiota. Front. Cell. Infect. Microbiol..

[27] Markowiak-Kopeć P., Śliżewska K. (2020). The effect of probiotics on the production of short-chain fatty acids by human intestinal microbiome. Nutrients.

[28] Lau A.S.Y., Yanagisawa N., Hor Y.Y., Lew L.C., Ong J.S., Chuah L.O., Lee Y.Y., Choi S.B., Rashid F., Wahid N. (2018). *Bifidobacterium longum* BB536 alleviated upper respiratory illnesses and modulated gut microbiota profiles in Malaysian pre-school children. Benef. Microbes.

[29] Ramirez-Farias C., Slezak K., Fuller Z., Duncan A., Holtrop G., Louis P. (2008). Effect of inulin on the human gut microbiota: Stimulation of *Bifidobacterium adolescentis* and *Faecalibacterium prausnitzii*. Br. J. Nutr..

[30] Louis P., Young P., Holtrop G., Flint H.J. (2010). Diversity of human colonic butyrate-producing bacteria revealed by analysis of the butyryl-CoA:acetate CoA-transferase gene. Environ. Microbiol..

[31] Zhou L., Zhang M., Wang Y., Dorfman R.G., Liu H., Yu T., Chen X., Tang D., Xu L., Yin Y. (2018). *Faecalibacterium prausnitzii* produces butyrate to maintain Th17/Treg balance and to ameliorate colorectal colitis by inhibiting histone deacetylase 1. Inflamm. Bowel Dis..

[32] Zhuang M., Shang W., Ma Q., Strappe P., Zhou Z. (2019). Abundance of probiotics and butyrate-production microbiome manages constipation via short-chain fatty acids production and hormones secretion. Mol. Nutr. Food Res..

[33] Zagato E., Pozzi C., Bertocchi A., Schioppa T., Saccheri F., Guglietta S., Fosso B., Melocchi L., Nizzoli G., Troisi J. (2020). Endogenous murine microbiota member *Faecalibaculum rodentium* and its human homologue protect from intestinal tumour growth. Nat. Microbiol..

[34] Zhang Q., Li G., Zhao W., Wang X., He J., Zhou L., Zhang X., An P., Liu Y., Zhang C. (2024). Efficacy of *Bifidobacterium animalis* subsp. lactis BL-99 in the treatment of functional dyspepsia: A randomized placebo-controlled clinical trial. Nat. Commun..

[35] Touchefeu Y., Duchalais E., des Varannes S.B., Alameddine J., Mirallie E., Matysiak-Budnik T., Le Bastard Q., Javaudin F., Rimbert M., Jotereau F. (2020). Concomitant decrease of double-positive lymphocyte population CD4CD8αα and Faecalibacterium prausnitzii in patients with colorectal cancer. Eur. J. Gastroenterol. Hepatol..

[36] Gupta S.K., Vyavahare S., Blanes I.L.D., Berger F., Isales C., Fulzele S. (2023). Microbiota-derived tryptophan metabolism: Impacts on health, aging, and disease. Exp. Gerontol..

[37] Laursen M.F., Sakanaka M., von Burg N., Mörbe U., Andersen D., Moll J.M., Pekmez C.T., Rivollier A., Michaelsen K.F., Mølgaard C. (2021). *Bifidobacterium* species associated with breastfeeding produce aromatic lactic acids in the infant gut. Nat. Microbiol..

[38] Henrick B.M., Rodriguez L., Lakshmikanth T., Pou C., Henckel E., Arzoomand A., Olin A., Wang J., Mikes J., Tan Z. (2021). Bifidobacteria-mediated immune system imprinting early in life. Cell.

[39] Sakurai T., Odamaki T., Xiao J.-Z. (2019). Production of indole-3-lactic acid by *Bifidobacterium* strains isolated fromhuman infants. Microorganisms.

[40] Lamas B., Richard M.L., Leducq V., Pham H.-P., Michel M.-L., Da Costa G., Bridonneau C., Jegou S., Hoffmann T.W., Natividad J.M. (2016). CARD9 impacts colitis by altering gut microbiota metabolism of tryptophan into aryl hydrocarbon receptor ligands. Nat. Med..

[41] Hendrikx T., Duan Y., Wang Y., Oh J.-H., Alexander L.M., Huang W., Stärkel P., Ho S.B., Gao B., Fiehn O. (2019). Bacteria engineered to produce IL-22 in intestine induce expression of REG3G to reduce ethanol-induced liver disease in mice. Gut.

[42] Zelante T., Iannitti R.G., Cunha C., De Luca A., Giovannini G., Pieraccini G., Zecchi R., D’Angelo C., Massi-Benedetti C., Fallarino F. (2013). Tryptophan catabolites from microbiota engage aryl hydrocarbon receptor and balance mucosal reactivity via interleukin-22. Immunity.

[43] Scott S.A., Fu J., Chang P.V. (2020). Microbial tryptophan metabolites regulate gut barrier function via the aryl hydrocarbon receptor. Proc. Natl. Acad. Sci. USA.

[44] Bröer S. (2023). Intestinal amino acid transport and metabolic health. Annu. Rev. Nutr..

[45] Xue C., Li G., Zheng Q., Gu X., Shi Q., Su Y., Chu Q., Yuan X., Bao Z., Lu J. (2023). Tryptophan metabolism in health and disease. Cell Metab..

[46] Siska P.J., Jiao J., Matos C., Singer K., Berger R.S., Dettmer K., Oefner P.J., Cully M.D., Wang Z., QuinnIii W.J. (2021). Kynurenine induces T cell fat catabolism and has limited suppressive effects in vivo. eBioMedicine.

[47] Wieckiewicz M., Martynowicz H., Lavigne G., Lobbezoo F., Kato T., Winocur E., Wezgowiec J., Danel D., Wojakowska A., Mazur G. (2023). An exploratory study on the association between serotonin and sleep breathing disorders. Sci. Rep..

[48] Walden K.E., Moon J.M., Hagele A.M., Allen L.E., Gaige C.J., Krieger J.M., Jager R., Mumford P.W., Pane M., Kerksick C.M. (2023). A randomized controlled trial to examine the impact of a multi-strain probiotic on self-reported indicators of depression, anxiety, mood, and associated biomarkers. Front. Nutr..

[49] Pinto-Sanchez M.I., Hall G.B., Ghajar K., Nardelli A., Bolino C., Lau J.T., Martin F.-P., Cominetti O., Welsh C., Rieder A. (2017). Probiotic *Bifidobacterium longum* NCC3001 reduces depression scores and alters brain activity: A pilot study in patients with irritable bowel syndrome. Gastroenterology.

[50] Su X., Gao Y., Yang R. (2022). Gut microbiota-derived tryptophan metabolites maintain gut and systemic homeostasis. Cells.

[51] Koper J.E., Troise A.D., Loonen L.M., Vitaglione P., Capuano E., Fogliano V., Wells J.M. (2022). Tryptophan supplementation increases the production of microbial-derived AhR agonists in an in vitro simulator of intestinal microbial ecosystem. J. Agric. Food Chem..

[52] Tian P., Chen Y., Zhu H., Wang L., Qian X., Zou R., Zhao J., Zhang H., Qian L., Wang Q. (2022). *Bifidobacterium breve* CCFM1025 attenuates major depression disorder via regulating gut microbiome and tryptophan metabolism: A randomized clinical trial. Brain Behav. Immun..

[53] Sharma V., Rodionov D.A., Leyn S.A., Tran D., Iablokov S.N., Ding H., Peterson D.A., Osterman A.L., Peterson S.N. (2019). B-vitamin sharing promotes stability of gut microbial communities. Front. Microbiol..

[54] van Elsas J.D., Chiurazzi M., Mallon C.A., Elhottova D., Kristufek V., Salles J.F. (2012). Microbial diversity determines the invasion of soil by a bacterial pathogen. Proc. Natl. Acad. Sci. USA.

