# Supplementary material for: The Impact of Fermented Milk Products Containing Bifidobacterium longum BB536 on the Gut Environment: A Randomized Double-Blind Placebo-Controlled Trial
Source: Nutrients. 2024 Oct 22;16(21):3580. doi: 10.3390/nu16213580 (PMC11547261; doi:10.3390/nu16213580)
Supplement: Supplementary file 1 [file nutrients-16-03580-s001.zip › Supplementary Figures_241021.pptx]

## Slide 1
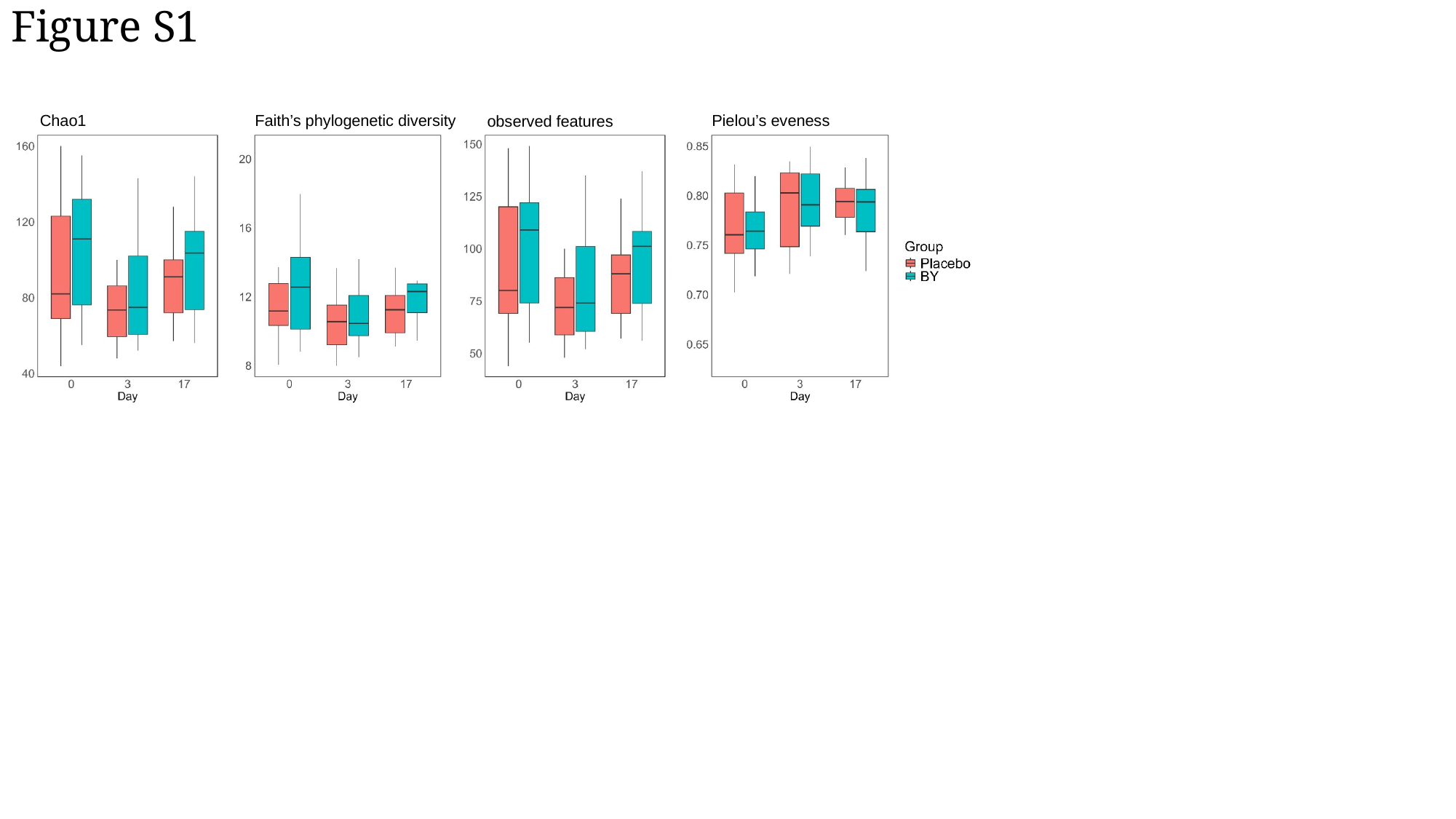

# Figure S1
Faith’s phylogenetic diversity
Pielou’s eveness
observed features
Chao1

## Slide 2
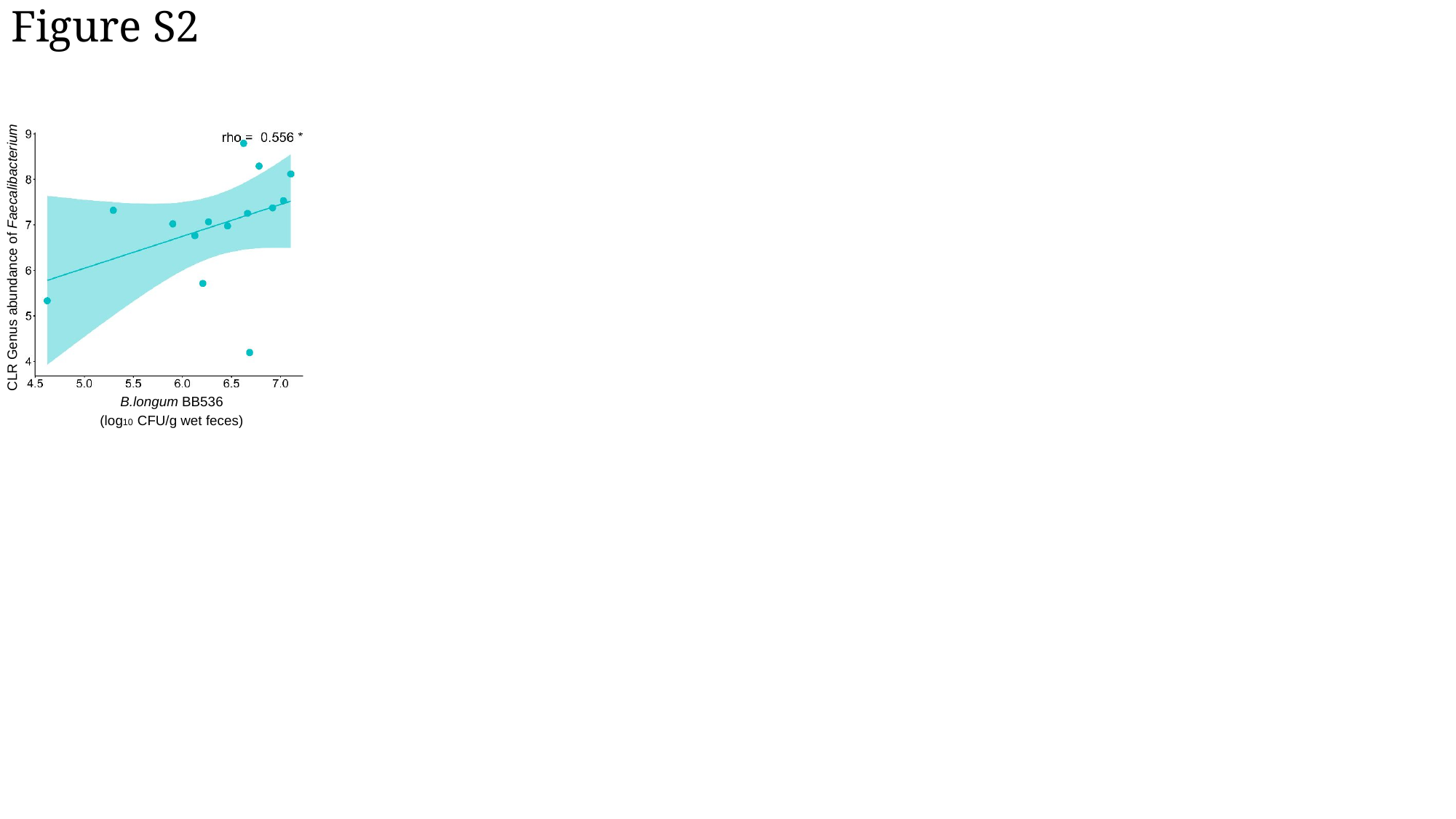

# Figure S2
CLR Genus abundance of Faecalibacterium
B.longum BB536
(log10 CFU/g wet feces)

## Slide 3
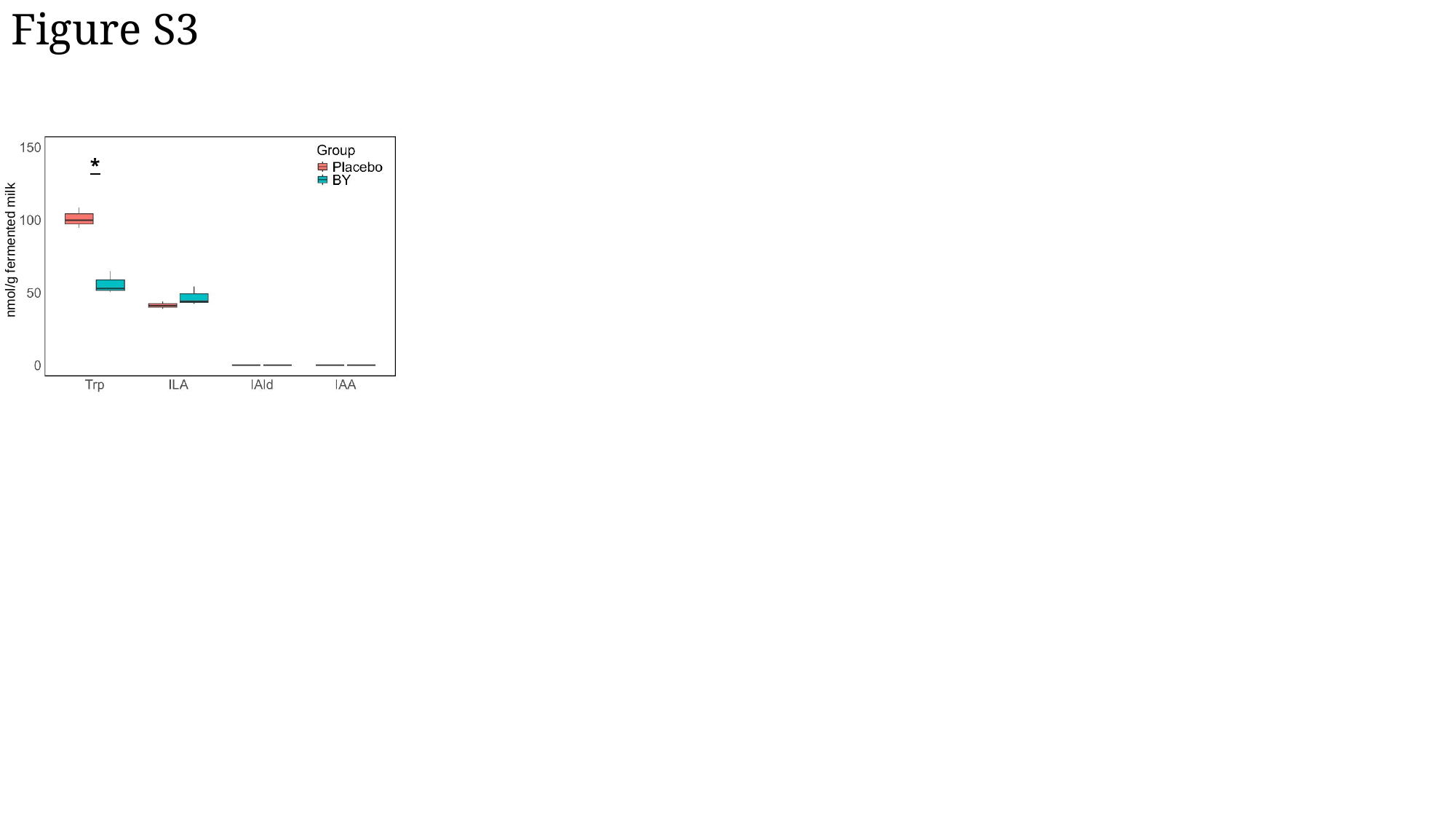

Figure S3
*
nmol/g fermented milk
